# Evolution and functional characterization of pectate lyase PEL12, a member of a highly expanded *Clonostachys rosea* polysaccharide lyase 1 family

**DOI:** 10.1186/s12866-018-1310-9

**Published:** 2018-11-07

**Authors:** Lea Atanasova, Mukesh Dubey, Marica Grujić, Mikael Gudmundsson, Cindy Lorenz, Mats Sandgren, Christian P. Kubicek, Dan Funck Jensen, Magnus Karlsson

**Affiliations:** 1 Forest Mycology and Plant Pathology, Swedish University of Agricultural Sciences, P.O. Box 7026, SE-75007 Uppsala, Sweden; 2 Research division of Biochemical Technology, Institute of Chemical, Environmental and Biological Engineering, Vienna University of Technology, Gumpendorferstrasse 1a, 1060 Vienna, Austria; 3 Molecular Sciences, Swedish University of Agricultural Sciences, P.O. Box 7015, SE-75007 Uppsala, Sweden; 4 Institute of Food Technology, University of Natural Resources and Life Sciences, Muthgasse 11, 1190 Vienna, Austria; 5 Present address: Steinschötelgasse 7, 1100, Vienna, Austria

**Keywords:** Pectin, *Clonostachys rosea*, *Trichoderma reesei*, Phylogeny, Enzyme, Plant biomass degradation

## Abstract

**Background:**

Pectin is one of the major and most complex plant cell wall components that needs to be overcome by microorganisms as part of their strategies for plant invasion or nutrition. Microbial pectinolytic enzymes therefore play a significant role for plant-associated microorganisms and for the decomposition and recycling of plant organic matter. Recently, comparative studies revealed significant gene copy number expansion of the polysaccharide lyase 1 (PL1) pectin/pectate lyase gene family in the *Clonostachys rosea* genome, while only low numbers were found in *Trichoderma* species. Both of these fungal genera are widely known for their ability to parasitize and kill other fungi (mycoparasitism) and certain species are thus used for biocontrol of plant pathogenic fungi.

**Results:**

In order to understand the role of the high number of pectin degrading enzymes in *Clonostachys*, we studied diversity and evolution of the PL1 gene family in *C. rosea* compared with other Sordariomycetes with varying nutritional life styles. Out of 17 members of *C. rosea* PL1, we could only detect two to be secreted at acidic pH. One of them, the pectate lyase *pel12* gene was found to be strongly induced by pectin and, to a lower degree, by polygalacturonic acid. Heterologous expression of the PEL12 in a PL1-free background of *T. reesei* revealed direct enzymatic involvement of this protein in utilization of pectin at pH 5 without a requirement for Ca^2+^. The mutants showed increased utilization of pectin compounds, but did not increase biocontrol ability in detached leaf assay against the plant pathogen *Botrytis cinerea* compared to the wild type.

**Conclusions:**

In this study, we aimed to gain insight into diversity and evolution of the PL1 gene family in *C. rosea* and other Sordariomycete species in relation to their nutritional modes. We show that *C. rosea* PL1 expansion does not correlate with its mycoparasitic nutritional mode and resembles those of strong plant pathogenic fungi. We further investigated regulation, specificity and function of the *C. rosea* PEL12 and show that this enzyme is directly involved in degradation of pectin and pectin-related compounds, but not in *C. rosea* biocontrol.

**Electronic supplementary material:**

The online version of this article (10.1186/s12866-018-1310-9) contains supplementary material, which is available to authorized users.

## Background

Pectin is a structural heteropolysaccharide present in the primary cell walls and in the middle lamella of terrestrial plants. The functions of pectin in cell walls are diverse and include plant growth and development, morphogenesis, defense, cell adhesion, cell wall structure, cellular expansion, porosity, ion binding, hydration of seeds, leaf abscission and fruit development, among others [1, 2]. In nature, pectic structures are extremely diverse, depending on the origin of the plant and on the plant tissue. Three pectic structural elements, homogalacturonan, rhamnogalacturonan-I and substituted galacturonans have been isolated from primary cell walls, are structurally characterized [3] and contain different degree of their main component, D-galacturonic acid. Substituted galacturonans are characterized by the presence of saccharide appendant residues branching from a backbone of D-galacturonic acid residues [4], whereas in rhamnogalacturonan I, the galacturonic acid residues are partly substituted by α-1-2 linked rhamnose residues. In addition, several side chains containing sugars, such as xylose, arabinose, glucose, fucose, mannose or galactose, have been found to be linked to the main backbone structure [5]. The galacturonic acid moieties in the backbone can also be esterified on the carboxylic acid moiety by methyl groups.

Polysaccharide lyases (PL) are a group of enzymes that cleave uronic acid-containing polysaccharide chains via a β-elimination mechanism to generate an unsaturated hexenuronic acid residue and a new reducing end [6]. The CAZy database (http://www.cazy.org; [7] has classified these enzymes to families and subfamilies to reflect their structural features and substrate specificities. The database currently comprises 21 PL families, from which PL1, PL3 and PL9 contain fungal pectin/pectate lyases [8]. Based on the methylation degree of the homogalacturan backbone of pectin, pectin and pectate lyases either cleave methylated or demethylated regions of pectin, respectively. Both enzymes share a conserved structure in a parallel β-helix and are grouped together in polysaccharide lyase family 1 (PL1; EC 4.2.2.2, EC 4.2.2.9 and EC 4.2.2.10.) [7].

Pectate lyases cleave glycosidic linkages preferentially in polygalacturonic acid, forming an unsaturated product (Δ-4,5-D-galacturonate) through a transelimination reaction. Contrary to pectin lyases they have an absolute requirement of Ca^2+^ ions and are classified as endo-pectate lyases (EC 4.2.2.2) that acts towards the substrate in a random way, and exo-pectate lyases (EC 4.2.2.9) that catalyze substrate cleavage from the non-reducing end [9]. Pectin lyases catalyze the random cleavage of pectin, preferentially highly esterified pectin, producing unsaturated methyloligogalacturonates [9]. The two types of lyases display essential differences in their active site, which is responsible for substrate binding and optimum pH. Pectin lyases have optimal activity at pH around 5.5, whereas pectate lyases favor higher pH optima around 8.5 [10]. However, Pedolli et al. [9] showed that many fungal lyases have their optimal activity at acid and neutral pH, while those from bacteria are more active at alkaline pH. Nevertheless, it is still an enigma why pectate lyases are more active at a basic pH when the pH of the plant cell wall is acidic [11].

Microorganisms produce plant cell wall-degrading enzymes as part of their strategies for plant invasion or plant degradation. Pectic enzymes can cause plant tissue maceration, cell lysis and modification of the cell wall structure, allowing other depolymerasing enzymes to act on their respective substrates [12]. Pectic enzymes therefore play a crucial role in microbial plant biomass degradation. Several fungal pectin/pectate lyases from a variety of microbial species have been functionally characterized, such as the saprotrophic/opportunistic *Aspergillus niger* [13–16], *A. oryzae* [16, 17], *Penicillium griseoroseum* [18], *P. occitanis* [19], and the phytopathogenic fungi *Glomerella cingulata* [20] *Colletotrichium gloeosporioides* [21], *C*. *lindemuthianum* [22] and *Botrytis cinerea* [23]. Recently, comparative genome analysis of *Clonostachys rosea* (Hypocreales, Bionectriaceae) revealed that PL1 is one of the most significantly expanded gene families in this ubiquitous mycoparasitic fungus compared to nine closely related Sordariomycetes [24]. *C. rosea* is a filamentous fungus that colonizes living plants thereby activating induced resistance, competes with other fungi for space and nutrients in the soil and the rhizosphere and directly parasitizes on other fungi and nematodes, and is thus of interest as a biological control agent [25, 26]. Taxonomically, *C. rosea* belongs to the same order (Hypocreales) but a different family (Bionectriaceae) than the more studied *Trichoderma* spp. mycoparasitic species (Hypocreaceae) [24]. In contrast to *C. rosea*, *Trichoderma* spp. have none or only few PL1 genes in their genomes (*T. reesei* and *T. virens* none, *T. atroviride* two genes), more similar to the saprotrophic *Neurospora crassa* [24].

In this study, we aimed to gain more insight into diversity and evolution of the PL1 gene family in *C. rosea* and other Sordariomycete species in relation to their nutritional modes. We investigated the evolution of PL1 enzymes in *C. rosea* using genome wide analysis of PL1 genes, and specificity and function of the *pel12* gene coding for the only pectate lyase protein that is secreted in acidic pectic conditions.

## Results

### Genome wide analysis of polysaccharide lyases in *C. rosea*

**Fig. 1:**
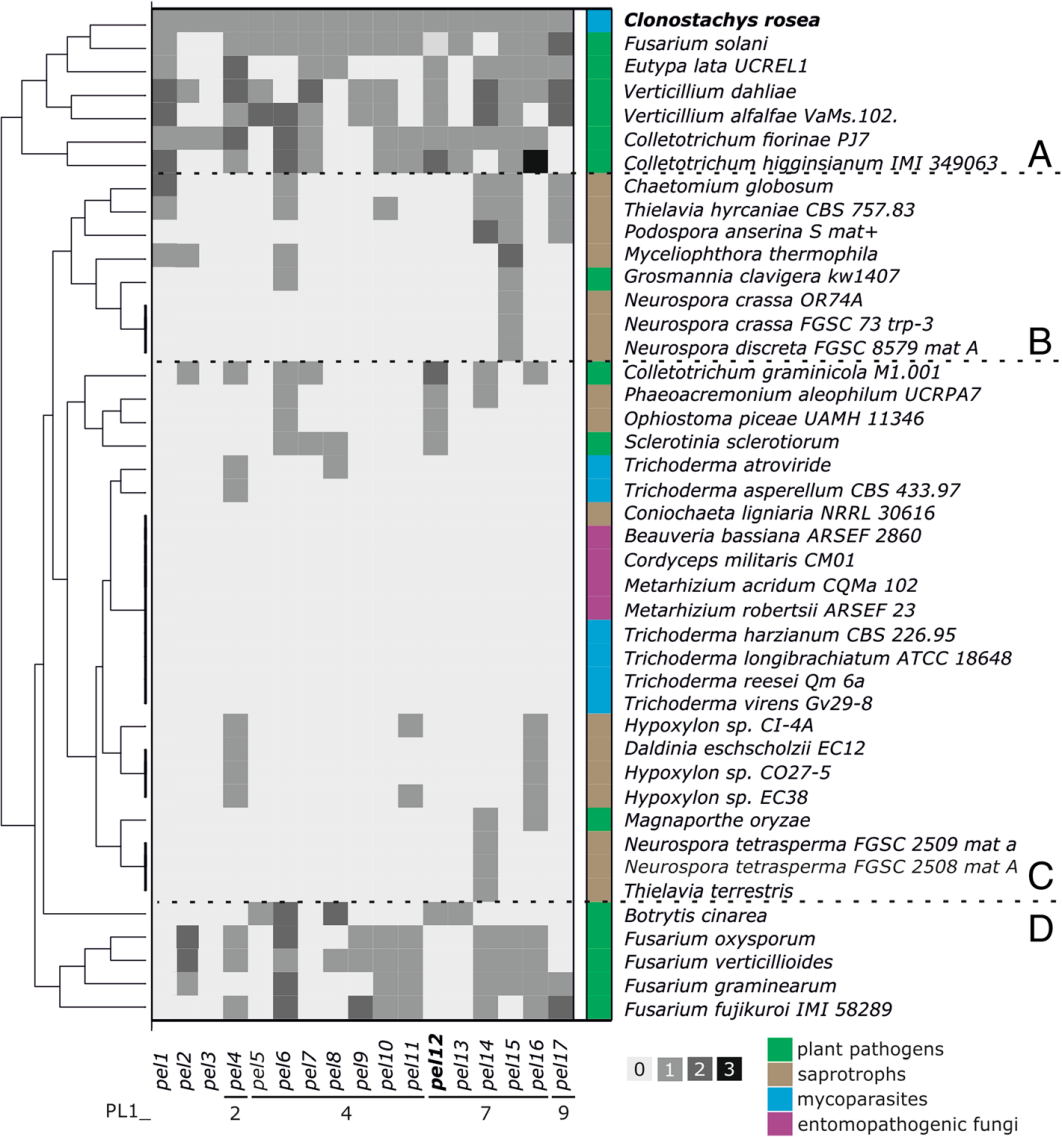
Hierarchical clustering based on PL1 gene content in Sordariomycetes and *Botrytis cinerea* and *Sclerotinia sclerotiorum* (Leotiomycetes). The clusters are marked with letters A to D. The squares colored with four different shades of gray represent the number of *C. rosea* orthologs per species. The color annotation on the right marks the nutritional mode of fungi

Sequence similarity and domain screening confirmed the presence of 17 PL1 family genes in the genome of *C. rosea* IK726. We named *C. rosea* PL1 genes and their according proteins *pel1*/PEL1 to *pel17*/PEL17 as listed in Table 1. The Cazy modules and subfamilies delimitation revealed that *C. rosea* PL1 contained four subfamilies; 2, 4, 7 and 9, while three genes could not be assigned to any subfamilies (Table 1 and Fig. 1). Out of seven genes from the PL1_4 subfamily, two were predicted to carry fungal-type CBM1 at the C-terminal, whereas the predicted PEL17 sequence from the PL1_9 subfamily contained a CBM1 at the N-terminal. Besides the PL1 domain, PEL3 was predicted to contain a pectinesterase domain (pfam01095) belonging to the CE8 pectin methylesterases (EC 3.1.1.11) family. As for the other PL families, ten PL3 genes (pectate lyases), three PL4 genes (rhamnogalacturonan lyases), and one gene each of PL20 and PL9 were further identified in *C. rosea*.

**Table 1:** PL genes in the *C. rosea* genome with assigned protein IDs, gene name, PL families, additional domains and subfamily identification, known activity based on Cazy database, list of crystal structures related to the PL families and organisms for which 3D structures are available in the PDB database

*C. rosea* protein ID	Gene name	CAZy module(s) and subfamilies	Known Activities	PDB/3D structure accession nr.	3D structure status	Organism with known 3D structure
PL1 genes identified in *C. rosea*						
BN869_T00011408	*pel1*	PL1	pectate lyase (EC 4.2.2.2); exo-pectate lyase (EC 4.2.2.9); pectin lyase (EC 4.2.2.10)	1IDJ[A,B] 1IDK[A] 1QCX[A] 1PXZ[A,B]	parallel β-helix	*Aspergillus niger, Juniperus ashei*
BN869_T00008859	*pel2*					
BN869_T00000002	*pel3*	CE8-PL1				
BN869_T00000920	*pel4*	PL1_2				
BN869_T00008472	*pel5*	PL1_4				
BN869_T00010915	*pel6*					
BN869_T00006080	*pel7*					
BN869_T00010737	*pel8*					
BN869_T00008735	*pel9*					
BN869_T00007710	*pel10*	PL1_4-CBM1				
BN869_T00005779	*pel11*					
BN869_T00006915	*pel12*	PL1_7				
BN869_T00007653	*pel13*					
BN869_T00008627	*pel14*					
BN869_T00002081	*pel15*					
BN869_T00010228	*pel16*					
BN869_T00007566	*pel17*	CBM1-PL1_9				
Other PL gene families and PL subfamilies identified in *C. rosea*						
BN869_T00011160		PL20	endo-β-1,4-glucuronan lyase A (GlA) (EC 4.2.2.14)	2ZZJ[A]	putative beta-jelly roll fold	*Trichoderma reesei* NBRC 31329
BN869_T00008392		PL3_2	pectate lyase (EC 4.2.2.2)	1EE6[A] 3B4N[A,B] 3B8Y[A,B] 3B90[A,B]	parallel β-helix	*Bacillus* sp. KSM-P15, *Dickeya dadantii* 3937
BN869_T00003737						
BN869_T00012955						
BN869_T00007220						
BN869_T00010913						
BN869_T00013332						
BN869_T00000798						
BN869_T00011510						
BN869_T00010121						
BN869_T00013099						
BN869_T00002494		PL4_1	rhamnogalacturonan lyase (EC 4.2.2.23)	1NKG[A] 2XHN[A,B] 3NJV[A] 3NJX[A]	β-sandwich and β-sheet	*Aspergillus aculeatus* KSM 510
BN869_T00003115						
BN869_T00009470		PL4_3				
BN869_T00000818		PL9_3	pectate lyase (EC 4.2.2.2); exopolygalacturonate lyase (EC 4.2.2.9); thiopeptidoglycan lyase (EC 4.2.2.-)	1RU4[A]	parallel β-helix	*Dickeya dadantii* 3937

List of polysaccharide lyase (PL) genes in the *C. rosea* genome

Mining of the available Sordariomycetes genomes together with plant pathogenic Leotiomycetes *B. cinerea* and *S. sclerotiorum* revealed striking differences between the number of PL1 genes (Additional file 1: Table S2). From 213 genes sorted in 17 groups resembling *C. rosea* orthologs, *pel5*, *pel8, pel9* and *pel11* (PL1_4), *pel13* (PL1_7) and *pel3* had less than 10 orthologs in 42 species, whereas most PL1 genes in Sordariomycetes were found to be orthologs of *C. rosea pel6* (25 genes). *Pel3* orthologs were only found in *C. rosea* and *C. fiorinae*. Out of 43 genomes characterized, nine did not possess any PL1 genes at all. These represented some species of Hypocreales that parasitize on or kill other fungi (mycoparasites), such as species of the genus *Trichoderma* or those that feed on insects (entomopathogens), such as *Beauveria bassiana*, *Cordyceps militaris*, and *Metarhizium* spp*. *(Fig. 1, cluster C). Hierarchical clustering of the genes showed that expansions and contractions of the PL1 families in fungi might be evolutionary driven as taxonomically similar taxa contain similar blocks of orthologs, however the number of genes belonging to PL1 also strongly depended on the nutritional mode of the fungi. Species with very limited number of PL1 genes were found among saprotrophic Xylariales (*Daldinia* and *Hypoxylon* spp.) as well as in Sordariales; in the genus *Neurospora, Podospora* and *Thielavia terrestris* (Fig. 1, cluster B), whereas among the most expanded were, with an exception of of mycoparasitic *C. rosea,* plant pathogens of the order Glomerellales (*Verticillium* and *Glomerella*/*Colletotrichum* species), and hypocreaceous *Fusarium solani* (*Nectria haematococca*) (Fig. 1, cluster A). Interestingly, the rice blast pathogen *Magnaporthe oryzae* has a very small number of PL1 genes (only two genes), and the leotiomycetous plant pathogens *S. sclerotiorum* and *B. cinerea* have only four and seven genes belonging to PL1, respectively. Apart of *N. haematococca,* the plant pathogenic *Fusaria* (Hypocreales) comprised between nine and 11 PL1 orthologs (Fig. 1, cluster D), and clustered separately from other plant pathogens, all lacking orthologs of *pel1, pel5, pel7, pel12* and *pel13*. Among the currently published and up to date Sordariomycetes genome, *C. rosea* is the species with the highest PL1 gene number (17 genes).

### Acidic pectin conditions induce the secretion of *C. rosea* polygalacturonases, polysacharide lyases and arabinofuranosidases

We screened for *C. rosea* proteins secreted in acidic minimal medium (pH 5) containing 0.5% polygalacturonic acid and apple pectin, respectively. As a control, protein expression on glucose and sucrose was monitored. Abundant secretion of protein could be observed on pectin, however not on glucose and sucrose. A single clearly visible SDS-PAGE band at ca. 37 kDa was detected. Replicate samples were submitted for peptide mapping analysis using tandem mass spectroscopy (MS). The MS analysis detected a total 18 proteins with high confidence levels associated with the peptide sequence found and did not contain shared peptides (Table 2). Redundant proteins were automatically grouped and are not shown. The highest scoring protein was annotated as an endopolygalacturonase from the GH28 family*.* Two additional endopolygalacturonases from the same family were detected in the set. Furthermore, glucoamylase from GH15, two alpha-L-arabinofuranosidases from GH54, two PL1 pectin lyases and a β-fructosidase from GH34 were identified (Table 2). An unclassified exopolygalacturonase, a pectinesterase, a FAD (Flavin Adenine Dinucleotide)-dependent oxygenase and a series of peptidases were also secreted under the given conditions.

**Table 2:**
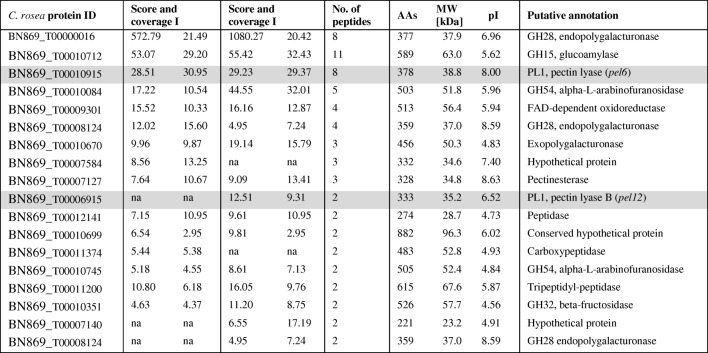
List of *C. rosea* proteins secreted on 0.5% pectin minimal medium in acidic condition (pH 4) obtained by tandem mass spectrometry. The protein score represents the sum of the scores of the individual peptides found in each replicate, whereas the coverage [in %] annotates the sequence covered by identified peptides. Number of distinct peptide sequences found in the protein group, sequence length (AAs), the calculated molecular weight (MW) and isoelectric point (pI) of the protein and putative annotation are listed. Members of PL1 are marked gray

Out of a total of 17 genes classified as PL1 and putatively involved in degradation of pectin, only two members were secreted during the cultivation of *C. rosea* in an acidic pectin medium, PEL6 and PEL12, respectively. These two proteins belong to subfamily 4 and 7 of PL1, respectively, and their theoretical molecular weight was estimated to be around 39 and 35 kDa, respectively. Interestingly, the *pel6* orthologs were found in most analyzed genomes (in 25 out of 43). *Pel12* orthologs were present in only 13 Sordariomycete species, in most plant pathogenic fungi and several saprotrophs with contracted PL1 families, but not in *M. oryzae* or in any *Fusarium* species, not even in *N. haematococca* with its highly expanded PL1 gene content (Fig. 1).

### Phylogenetic analysis reveals a *C. rosea pel12* gene paralog and a putatively new PL1 subfamily

**Fig. 2:**
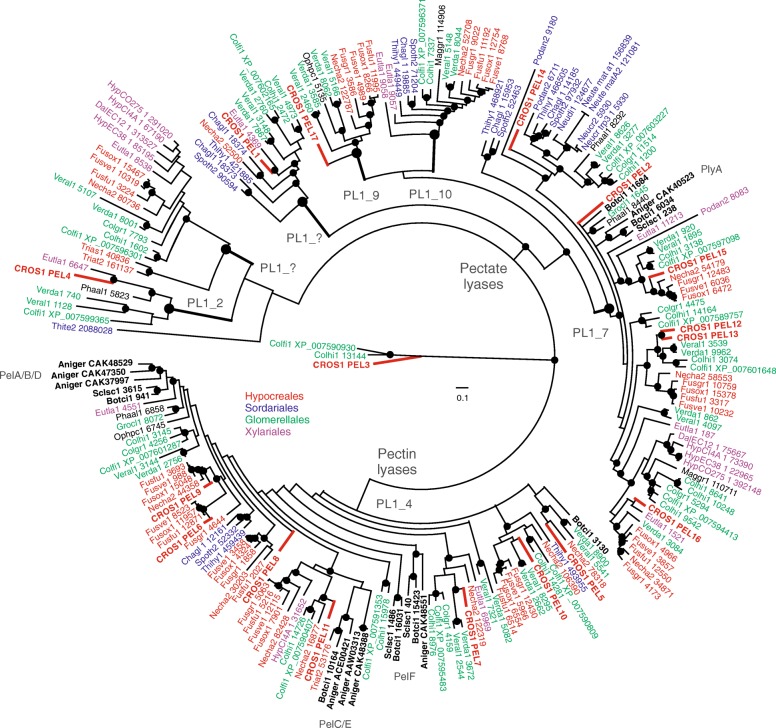
Phylogenetic relationships of PL1 proteins among Sordariomycetes, *A. niger* (Eurotiomycetes) and *S. sclerotiorum* and *B. cinerea* (Leotiomycetes). Predicted amino acid sequences were aligned by MUSCLE and were used to construct a Bayesian phylogenetic tree implemented in MrBayes. The marked nodes represent posterior probabilities greater or equal than 95%. The species abbreviations followed by genome protein IDs are given on the tree, the full species names are listed in the Additional file 1: Table S1. Included Leotiomyces and Eurotiomycetes are marked in bold black

Phylogenetic analysis of the PL1 protein family containing 41 Sordariomycete genomes, Leotiomycetes *B. cinerea* and *S. sclerotiorum* and *A. niger* (Eurotiomycetes) as an outgroup, revealed a clear separation of pectin and pectate lyase enzymes. Among pectate lyases, six supported clades were found, and based on sequence similarity with structurally characterized proteins [7] four of these clades were identified as PL1 subfamilies 2, 7, 9 and 10 (Fig. 2). Based on the well characterized *A. niger* PlyA protein [27, 28] the largest group was identified as subfamily 7, where six *C. rosea* PL1 paralogs were found. Interestingly, plyA was the only detected pectate lyase from *A. niger* and *S. sclerotiorum*. In contrast, Hypocrealean and Glomerellalean species contain many PL1_7 members and *C. rosea* PL1 proteins were overrepresented in this subfamily (PEL12 – PEL16). In addition, the *C. rosea* PL1_7 pectate lyase PEL12, secreted on pectin in acidic conditions (see above) and PEL13 revealed to be close protein paralogs (Fig. 2). The closest neighbors of these two *C. rosea* paralogs were Glomerellales pectate lyases, with the sister branch of orthologs from *Fusarium* spp. (Hypocreales). Moreover, PL1_9 and PL1_10 subfamilies contained proteins from Glomerellalean and Hypocrealean species, although PL1_10 also contained some members of Sordariales and at the basal position a member of Xylariales (Fig. 2). Furthermore, for two other pectate lyase clades, no sequence similarity to any so far characterized PL1 subfamily could be found. One of them includes the PEL1 pectate lyase and might, due to the statistical support of the clade, represent a putatively new subfamily among PL1.

The second large supported group represent pectin lyases, all part of PL1 subfamily 4. The analysis revealed many small supported subclades of which seven contained *C. rosea* members. Three of these groups included members of *A. niger* pectin lyases: PelF, PelC/E and PelA/B/D. Furthermore, *S. sclerotiorum* and *B. cinerea* (Leotiomycetes) PL1s were forming separated clades, but not at the basal position as evolutionary expected. Using the proteins of these species as outgroups, we speculate that the split of PL1 family in subgroups is likely older then subphilum Pezizomycotina.

Together with *C. rosea*, *T. atroviride* and *T. asperellum* were the only mycoparasites with PL1 proteins. The pectate lyase singlets of the latter two species grouped at the basal position in one of the putatively new PL1 subgroups. Pectin lyase from *T. atroviride*, however, was shown to be closely related to PelE from *A. niger* and *B. cinerea* (Fig. 2).

### Structural and regulatory divergences in *C. rosea* likely led to functional differentiation of PEL12 and its paralogs

**Fig. 3:**
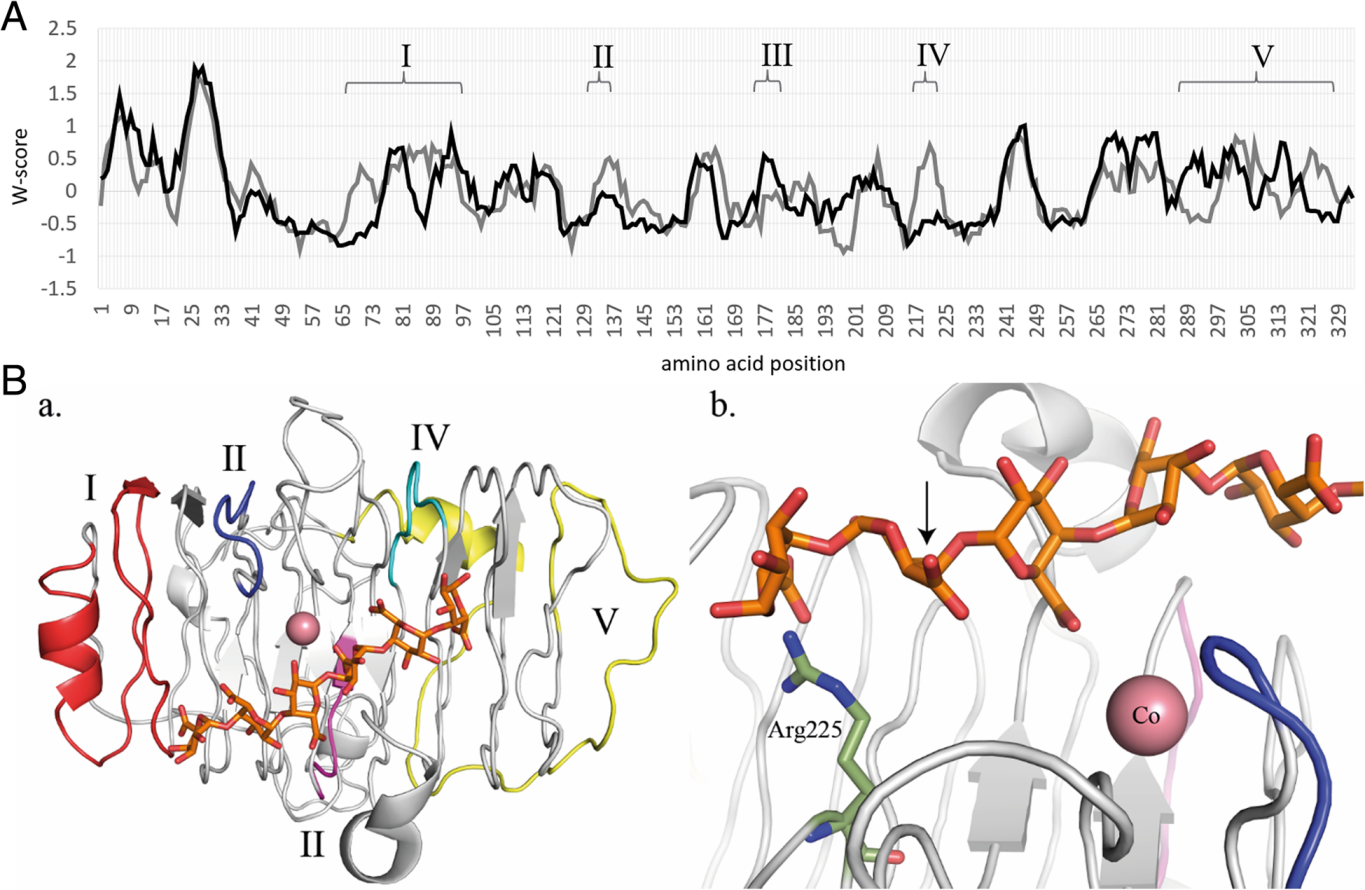
Reverse conservation analysis of PL1_7 orthologs revealed areas of putative functional divergence. **a** Amino acid conservation was estimated using Rate4Site, based on a MUSCLE alignment of fungal PL1_7 orthologs, and plotted as W mean scores in arbitrary units. The black and grey lines represent the closest orthologs in the PEL12/PEL13 and PEL2 clades (Additional file 2: Figure S1), respectively. **b** Cartoon representation of the PEL12 model. Defined regions are those that are mostly variable. I = pos. 67–98 (in red), II = pos. 131–137 (in blue), III = pos. 176–182 (in magenta), IV = pos. 217–223 (in cyan), V = pos. 287–327 (in yellow). The pentagalacturonate ligand is shown in orange sticks, and the two calcium ions positioned at two putative Ca^2+^ binding site, are shown in pink. In subfigure b. the catalytic base, Arg 225, is shown in green sticks. The hydrolytic cleavage of the polygalacturonic substrate will occur at the position denoted by the arrow. The pentagalacturonate and calcium ligands are extracted from a superposition of PDB:ID 3KRG

The phylogenetic analysis of PL1 proteins revealed that *C. rosea* PEL12 and its close paralogue PEL13 were members of the pectate lyase PL1_7 subfamily, together with orthologs from plant pathogenic fungi with highly expended PL1 families such as *Verticillium* and *Colletotrichum* (*Glomerella*) (Fig. 2). An alignment of PL1_7 protein sequences was further analyzed to identify divergent amino acid sites and regions that may indicate functional differentiation between PL1_7 members (Additional file 2: Figure S1). Reverse Conservation Analysis (RCA) was applied to reveal conserved and variable regions between the PEL12/PEL13 clade and the closely related PEL2 clade. Five regions were identified that displayed signs of functional divergence; high variation in one clade in combination with low variation in the other clade (Fig. 3a). Regions I and V contained the *N-* and *C*-terminal flanks of the main β-helix structure, respectively. Regions II, III and IV were shorter and comprise loops in the central β-sheet core. Homologous structures and homology modelling suggested that the linear pectin polymer substrate of PL1 enzymes likely binds along one face of the β-helix axis. The active site where the enzymatic β-elimination cleavage of galacturonate occurs is located between the catalytic base (putatively arginine Arg225 in PEL12) and two Ca^2+^ ions (Fig. 3b). The pentagalacturonate ligand and the two putative calcium ions were extracted from a homologous *Bacillus subtilis* pectate lyase crystal structure (PDB:ID 3KRG) to visualize functional areas. Regions III and IV were located close to the active site but on the opposite face from the binding cleft of the β-helix.

**Fig. 4:**
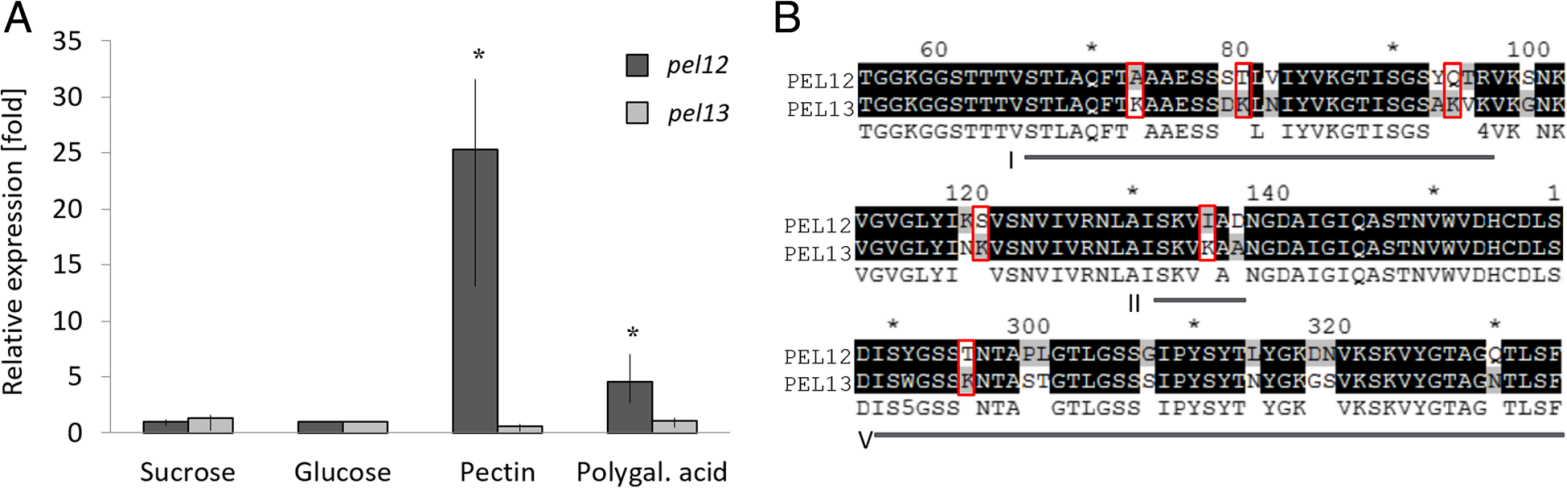
**a** Differential gene expression of the *pel12* (BN869_T0006915) and *pel13* (BN869_T00007653) genes in *C. rosea*, induced by 0.5% sucrose, glucose, pectin or polygalacturonic acid. Asterisks indicate significantly (*P* ≤ 0.05) higher expression compared with the glucose control treatment. **b** Mutational shifts from non-charged amino acids to positively charged lysine (red squares) in the PEL12 and PEL13 protein sequences. Roman numbers mark the evolutionary variable regions I, II and V detected between the closest paralogs (see Fig. 3). Asterisks mark next ten amino acids

PEL12 and its paralog PEL13 shared 78% of identity on the amino acid level, but contrary to PEL12, PEL13 was not detected among the proteins secreted on apple pectin at pH 4 (Table 2). Gene expression analysis of *pel12* and *pel13* on 0.5% glucose, sucrose, pectin and polygalacturonic acid revealed major differences in their transcriptional responses. Expression of *pel12* was strongly induced on apple pectin and polygalacturonic acid whereas its paralog was transcribed but not differentially regulated in these conditions (Fig. 4). The protein sequence alignment showed several mutational shifts from non-charged amino acids in PEL12 to positively charged lysine in the paralog, mostly located in regions I, II and V (Fig. 4) that displayed signs of functional divergence between the PEL12/PEL13 and PEL2 clades. Particularly, regions I and II were located in the substrate entry/exit areas where differences in charge distribution might impact substrate selectivity [29].

Furthermore, the promoter sequences (1 kb upstream of the start codon) of *pel12* and *pel13* displayed major differences in their nucleotide sequences. Analyses with the JASPAR CORE fungi tool [30] showed that HAP2 binding sites were commonly present in both promoter regions. HAP2 acts as a component of the CCAT-binding factor, a transcriptional activator that binds to the upstream activation site (UAS2) of the CYC1 gene and other genes involved in mitochondrial electron transport and activate their expression in *Saccharomyces cerevisiae*. In contrast, putative binding sites for pH-response transcription factor pacC/RIM101, activator of stress genes ASG1, regulatory protein CAT8, transcription factor PDR8; transcription factor MBP1, regulatory protein SWI6 and regulator of drug sensitivity RDS1 were only found in the *pel12* promoter, whereas Ty transcription activator TEC1, pre-rRNA-processing protein FHL1 and arginine metabolism regulation protein ARGR1 binding sites were only detected in the *pel13* promoter.

### PEL12 induces utilization of pectin-related compounds in *T. reesei pel12*OE mutants

**Fig. 5:**
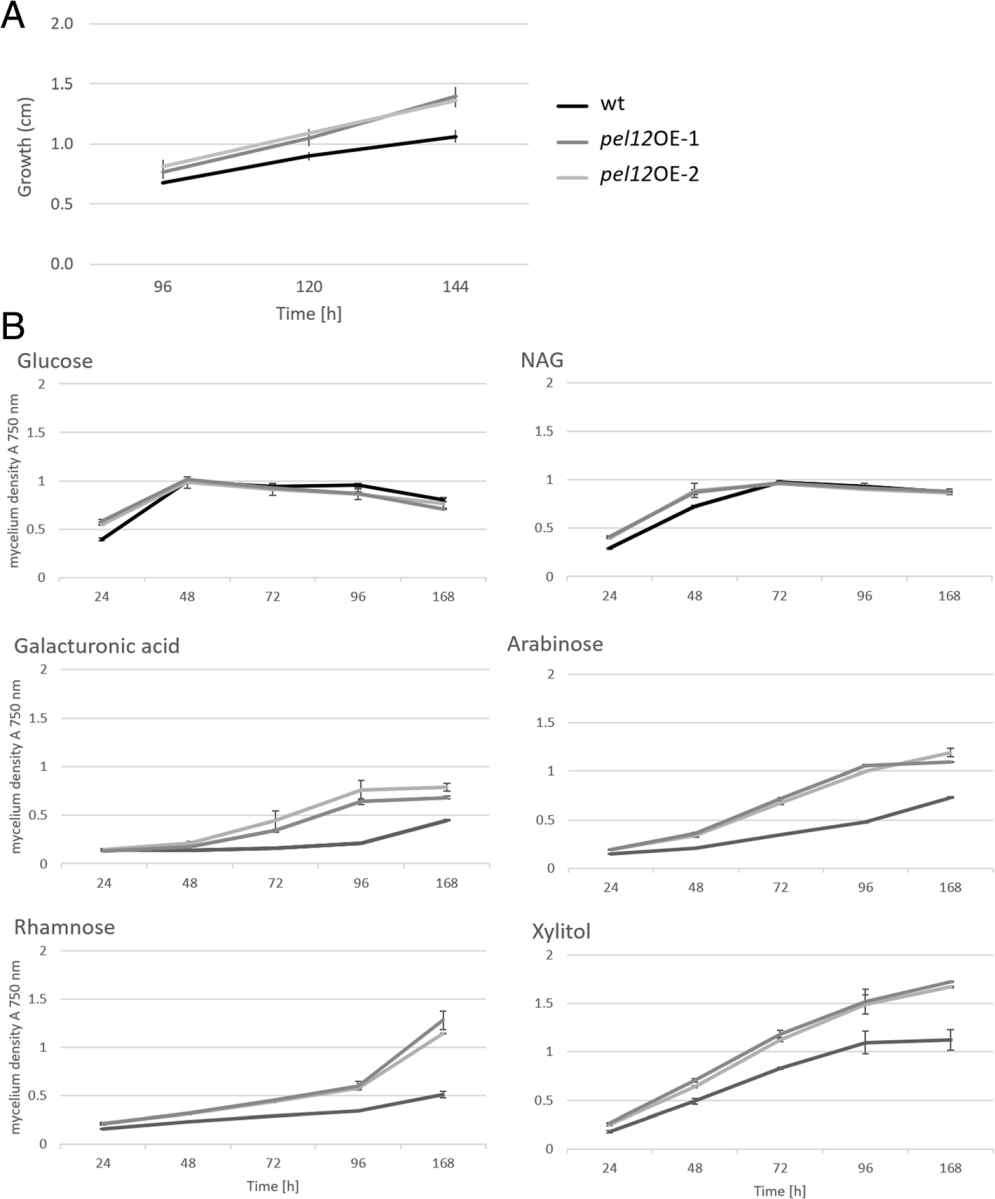
Utilization of carbon sources in *T. reesei* QM 9414 and *pel12*OE mutants. **a** Growth of strains on 1% agarose supplemented with 2% pectin. **b** Growth rates of strains on selected carbon sources assessed by BIOLOG Phenotypic assay for filamentous fungi. The cultures were incubated at 28 °C in darkness

The PEL12 protein was overexpressed in *T. reesei* QM 9414 (WT) under the constitutive enolase promoter and *Cel7a* terminator. The gene integration into *T. reesei* genome was verified by the *pel12* overexpression primers (Additional file 1: Table S2). As mentioned above, the expression of *C. rosea* PEL12 was detected as a 37 kDa band on a SDS-PAGE gel. The overexpressed PEL12 protein was detected at the correct size in *T. reesei pel12*OE-1 and *pel12*OE-2 mutants but not in the WT culture filtrates after 48 h of cultivation on 1% glucose (Additional file 2: Figure S2). Gene expression analysis confirmed a constitutive expression of *pel12* in *T. reesei* on inducing and repressing carbon sources (data not shown). The mutants exhibited significantly better growth on pectin medium and improved utilization of pectin compounds such as galacturonic acid, arabinose and rhamnose compared to the WT in the BIOLOG Phenotypic assay developed for filamentous fungi (Fig. 5). Growth rates on glucose, glycerol, galactose, fructose, and N-acetyl-D-glucosamine (NAG) were not altered. Indeed, several authors have proposed the existence of a regulatory system that activates the expression of a group of pectinolytic genes in response to galacturonic acid, and activates other pectinolytic genes in response to L-arabinose, L-rhamnose, ferulic acid and D-xylose [27, 31, 32]. Interestingly, it became evident that *T. reesei* utilizes galacturonic acid at a very low level (after water, at position 91 out of 95 carbon sources tested, see Additional file 2: Figure S3). In contrast, in the *pel12*OE mutants it was ranked among the first half of the best assimilated carbon sources (position 45). Xylitol, a sugar alcohol found in low concentrations in the fibers of many fruits and vegetables, was shown to be the best utilized carbon source by *pel12*OE mutants, and it elevated the growth of these strains much more than any of the best utilized compounds tested for the *T. reesei* WT (Additional file 2: Figure S3).

Enzymatic activity of heterologously expressed PEL12 was measured using the standard pectate and pectin lyase activity assay as described by Collmer et al. [12]. Concentrated crude protein extracts from *T. reesei* WT and *pel12*OE mutants were added to the substrates containing either polygalacturonic acid and CaCl_2_, or apple pectin, both at pH 8.5 and pH 5. The *pel12*OE-2 mutant supernatant yielded a linear activity of 0.02 μM min^− 1^ mL^− 1^ in first 10 min in the pectin substrate at pH 5, but not at pH 8.5 or in polygalacuronic acid. The WT and the *pel12*OE-1 mutant supernatants did not show any catalytic activity in any of repeated assays.

**Fig. 6:**
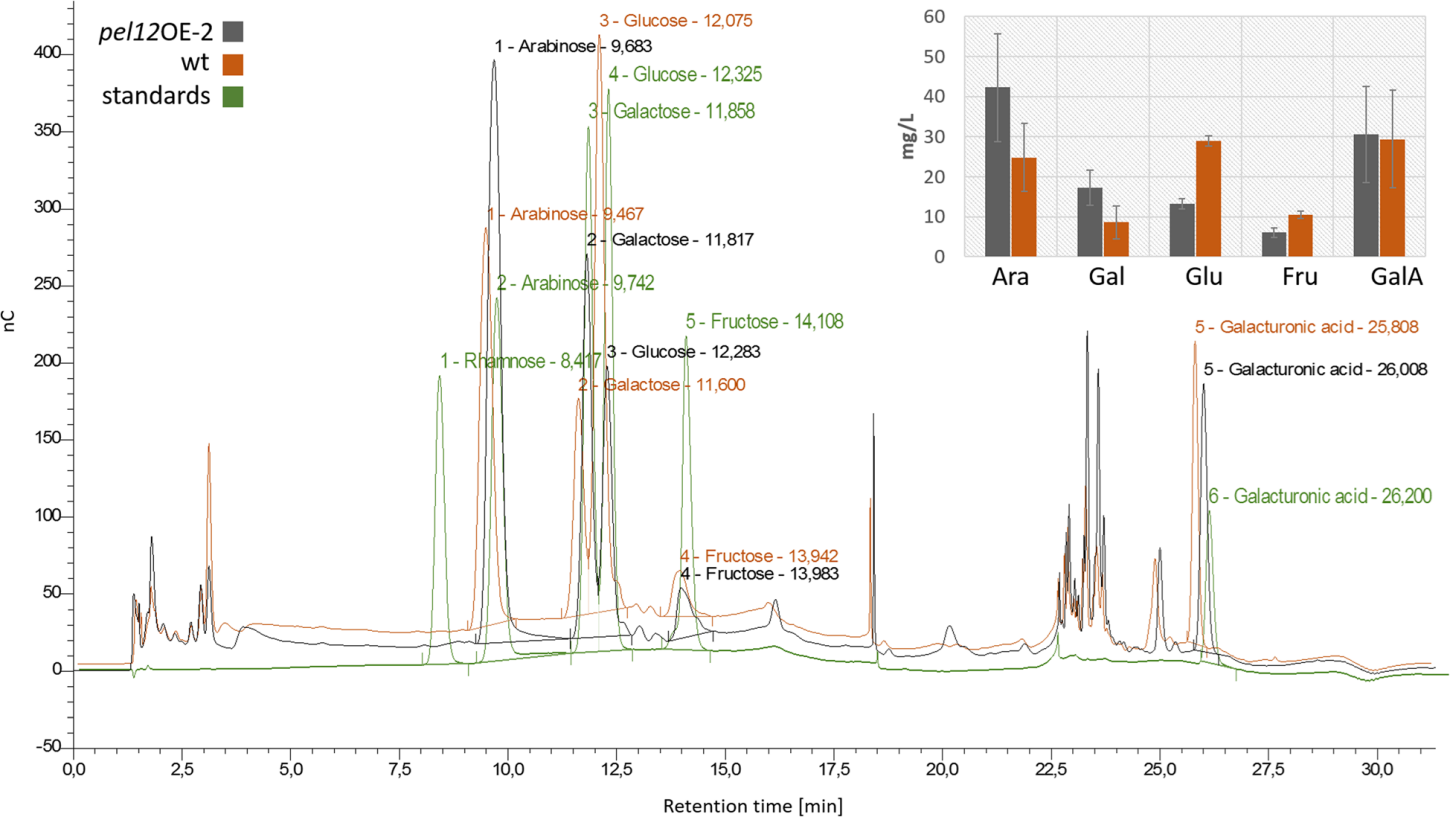
HPAEC-PAD analysis of *T. reesei* QM 9414 and the *pel12*OE-2 mutant supernatants collected after 72 h post inoculation on minimal medium supplemented with 1% apple pectin, pH 5. The bars represent the detected quantity of monosaccharides arabinose (Ara), galactose (Gal), glucose (Glu), fructose (Fru), and galacturonic acid (GalA) in the culture extracts. nC is the unit of electric charge measured in nano-Coulomb, meaning the charge transported by a constant current of one Ampere in one second

After cultivation of the strains on 1% apple pectin for 72 h, the presence of monosaccharides arabinose, glucose, galactose, fructose, and rhamnose as well as galacturonic acid in the *T. reesei* WT and the *pel12*OE-2 fermentation broths were quantified. Analysis was done by high-performance anion-exchange chromatography with pulsed amperometric gold electrode detection (HPAEC-PAD) paired with an anion exchange CarboPac PA1 column. Rhamnose was not released by either strain. In contrast, large differences in the ability to release arabinose, galactose and glucose were detected between the *T. reesei* WT and the *pel12*OE-2 mutant. The fermentation broth of *pel12*OE-2 contained around 15 mg L^− 1^ less glucose and also less fructose compared to the wild type, whereas arabinose and galactose were more abundant in the *pel12*OE-2 supernatant (Fig. 6). Furthermore, galacturonic acid content in the WT and the *pel12*OE-2 culture broths was very similar (Fig. 6). The occurrence of several peaks right before the galacturonic acid at a retention time of 22.5 to 25 min might indicate the presence of 4,5-unsaturated products released by the pectate/pectin cleavage of polygalacturonan usually detectable with the pectate/pectin assay at 232 nm. The *pel12*OE-2 mutant indeed showed an increase of these products in the culture filtrate compared to the WT.

### Deletion or overexpression of *pel12* does not affect the biocontrol potential of *C. rosea* or *T. reesei*

To determine the function of PEL12 we deleted the *pel12* gene from the *C. rosea* genome. Successful gene replacement in mitotically stable transformants was confirmed by PCR as described in our previous studies [33, 34]. Amplification of an expected size of PCR fragment in putative transformants, while no amplification in WT validated the successful deletion of *pel12* (Additional file 2: Figure S4A). Furthermore, RT-PCR experiments using primers specific to the *pel12* sequence demonstrated the complete loss of *pel12* transcript in each mutant (Additional file 2: Figure S4B). The deletion did not lead to any morphological differences in the mutants compared to the WT strain. Furthermore, the *pel12* deletion in *C. rosea* did not influence utilization of pectin compounds as tested with BIOLOG Phenotypic assay (Additional file 2: Figure S5). The biocontrol potential of *C. rosea* WT, *C. rosea* ∆*pel12* mutants, *T. reesei* WT and *T. reesei pel12*OE mutants against *B. cinerea* was evaluated using a detached *A. thaliana* leaf assay. All tested strains significantly reduced the lesion size caused by *B. cinerea*, but no quantitative or qualitative difference between the WT strains and their corresponding *pel12* mutants were detected (Additional file 2: Figure S6).

## Discussion

Despite the fact that *C. rosea* and *Trichoderma* spp. belong to different families (Bionectriaceae and Hypocreaceae, respectively) within the order Hypocreales, they share many commonalities in their lifestyle as rhizosphere-competent saprotrophs and necrotrophic mycoparasites. Because of these features, certain species of these genera are used as biological control agents in agricultural and horticultural crop production. However, recent comparative genomic studies of *C. rosea* and *Trichoderma* spp. [24, 35–37] revealed distinct differences in gene family evolution, indicative of fundamental differences in the life strategies of these fungi [38] with direct bearing for the implementation of biocontrol in agriculture. Karlsson et al. [24] identified the PL1 gene family to evolve under selection for increasing gene copy number in *C. rosea*, but not in *Trichoderma* spp., which prompted us to perform a more detailed investigation of these genes in *C. rosea*.

Analysis and hierarchical clustering of gene numbers in a set of different fungal genomes revealed a clear distinction of PL1 gene distribution related to nutritional mode; *Trichoderma* mycoparasites and entomopathogenic fungi have none or drastically reduced PL1 gene numbers, while PL1 gene numbers in plant pathogenic fungi such as *N. haematococca*, *Colletotrichum* and *Verticillium* are highly expanded. Plant pathogenic fungi use plant cell wall-degrading enzymes for penetration and nutrient acquisition, while plants have evolved strategies that allow them to detect and to defend against the attack of pathogens by producing inhibitors of these enzymes [22]. Pectic structures are also extremely diverse, depending on the plant and plant tissue. We can therefore speculate that the observed expansion of PL1 pectin/pectate lyase numbers in several plant pathogenic fungi is driven by selection for functional diversification with regard to regulation and substrate specificity. Our phylogenetic analysis of the PL1 gene family does not contradict this hypothesis, as we detected a high number of hierarchically organized subgroups and divisions, including two previously unknown PL1 subgroups, which may represent isozymes with particular properties. For example, significant differences between two fungal races of *C. lindemuthianum* were detected in terms of the expression of the *Clpnl2* gene encoding for pectin lyase 2, where the pathogenic race 1472 responded faster and with higher expression levels than the non-pathogenic race 0 [22]. Wijesundra et al. [39] reported that *C. lindemuthianum* race β secreted two forms of pectin lyase, having pI values of 8.2 and 9.7, respectively, when grown in culture with sodium polypectate or isolated *Phaseolus vulgaris* hypocotyl cell walls as the main carbon source. All the forms of pectin lyases showed maximum activity under alkaline conditions [39]. Furthermore, in *C. gloeosporioides*, a pectin/pectate lyase gene was considered important for infection of avocado fruit [40] and two pectin lyase genes, *pnl-1* and *pnl-2*, which encode for extracellular enzymes that differ both in their expression in pure culture and during infection, were isolated from *C. gloeosporioides* f. sp. *malvae* [21]. Expression of an exogenous pectinase in a pathogenic fungus may be sufficient to change the pathogenicity and the host: *C. magna*, a pathogen of cucurbits, was transformed with a pectate lyase gene from the avocado pathogen *C*. *gloesporioides* and became more aggressive on watermelon and avocado [40]. In contrast, expression of an endopolygalacturonase gene from *Fusarium oxysporum* f. sp. *Iycopersici* in strains of *F. oxysporum* f. sp. *melonis* did not induce any change in pathogenicity [41].

However, *C. rosea* is not a plant pathogenic fungus and hence the selective advantages for high numbers of PL1 genes in this species should be different from plant pathogenic fungi. *C. rosea* is reported to intimately interact with plants through root surface colonization [24, 42] and by penetrating epidermal cells [43]. This intimate interaction with plants can result in plant growth promotion [44] and induction of defense reactions in both wheat and tomato [44, 45], but it is difficult to argue how this superficial level of plant interaction can justify the existence of 17 PL1 genes in *C. rosea*. An alternative explanation is that PL1 pectin/pectate lyases are involved in saprotrophic utilization of plant organic matter in soil. It has been shown previously that germination and activity of *C. rosea* in soil depends on the presence of organic matter or root exudates [46]. It is plausible that degradation of pectic compounds in soil contributes to the versatile capacity of *C. rosea* to establish in soil and the rhizosphere.

The induction of several pectin-degrading enzymes, including the two different PL1 proteins PEL6 and PEL12, during growth on apple pectin shows that *C. rosea* indeed has the capacity to utilize pectin as a nutrient source. The phylogenetic analysis shows that PEL6 is a pectin lyase while PEL12 is a pectate lyase, suggesting their synergistic action during pectin degradation [27]. A closer inspection of the PL1 phylogenetic tree reveals an overrepresentation of PL1 genes from *C. rosea* and several plant pathogenic species in subgroup PL1_7, which include the secreted PEL12 pectate lyase. If the expansion of the PL1_7 subgroup is driven by selection for functional diversification, we would expect structural and regulatory differences between the *C. rosea* PL1_7 paralogs. An indication of differential regulation between PEL12 and its close paralog, PEL13, come from the fact that PEL12, but not PEL13, was detected in the culture broth of *C. rosea* grown on apple pectin. This was further confirmed at the transcriptional level as *pel12*, but not *pel13*, was induced by pectin and polygalacturonic acid. Instead, *pel13* was constitutively expressed during the investigated conditions showing that it is not a pseudogene. Substantial differences in the promoter regions of *pel12* and *pel13* provided an explanation for the differential expression. The presence of a putative binding motif for the pH-response transcription factor PacC in the *pel12* promoter is particularly interesting, as disruption of the *pacC* gene in *C. rosea* results in attenuated virulence against nematodes [47]. The serine protease gene *prC* in *C. rosea* also carry an inverted PacC binding motif in its promoter, and gene disruption of *prC* also results in attenuated virulence against nematodes [47, 48], thereby establishing a link between pH-responsiveness and nematode virulence in *C. rosea*. We also found indications of non-random structural changes between PL1_7 paralogs, localized in the predicted substrate entry/exit areas of the enzymes. Based on the homology modelling of the β-helix structure covering the main body of PEL12 with the carbohydrate binding site, we speculate that the variation between the PL1_7 paralogs results in charge-distribution alternations that may influence the affinity for methylated or non-methylated galacturonate, consequently leading to functional divergence.

Functional diversification of other carbohydrate-active enzymes has sometimes resulted in new biochemical functions or even loss of catalytic activity. For example, some chitinases have lost their catalytic activity but retained their chitin-binding capacity, resulting in lectin- or receptor-like proteins [49–51]. However, our data suggest that this is not the case with PEL12 in *C. rosea*, but rather that it constitutes an active pectate lyase. First, the homology modelling predicts the enzymatic β-elimination cleavage active site of PEL12 to occur between a catalytic arginine (Arg230) and a bound Ca^2+^ metal ligand, which is in accordance with the three-dimensional structures of five members of the pectate lyase superfamily: *Erwinia chrysanthemi* pectate lyase C (PELC) [52] and pectate lyase E (PELE) [53], *Bacillus subtilis* pectate lyase [54] and *A. niger* pectin lyase A (PLA) [10] and pectin lyase B (PLB) [55]. Secondly, overexpression of *pel12* in a PL1-free *T. reesei* background results in increased utilization of pectin and pectin-related carbon sources of the *T. reesei pel12*OE mutants, providing indirect evidence for pectinolytic activity of PEL12. Thirdly, detection of pectin/pectate lyase enzymatic activity in the supernatant, increased ability to release arabinose and galactose, and the presence of 4,5-unsaturated degradation products during growth on pectin by one of the *T. reesei pel12*OE mutants further support the conclusion that PEL12 is an active, pectinolytic enzyme.

Deletion of the *pel12* gene in *C. rosea* indicated that PEL12 is dispensable for growth on PDA and biocontrol of *B. cinerea* on *A. thaliana* leaves. It is possible that the remaining 16 PL1 genes, and the additional 15 pectinolytic genes (one GH20, 10 PL3, three PL4 and one PL9) that we detected in the *C. rosea* genome are responsible for masking an effect, but the lack of effect on biocontrol in *T. reesei* OE mutants indicates that PEL12 indeed has no role in the interaction of these mycoparasitic species and their fungal prey.

## Conclusions

In summary, we show that high numbers of PL1 genes are present in many plant pathogenic fungi, and the mycoparasite *C. rosea*. Specifically subgroup PL1_7 pectate lyases contain many *C. rosea* paralogs, and we provide indications for functional diversification between these paralogs. One member, *pel12*, encodes a functional pectinolytic enzyme that is induced by pectin and polygalacturonic acid, but is dispensable for biocontrol.

## Methods

### Strains and growth conditions used in this study

*C. rosea* strain IK726 and mutants derived from it, and *B. cinerea* strain B05.10 were maintained on potato dextrose agar (PDA) medium (Difco, Germany) at 25 °C. *T. reesei* strain QM9414 and mutants derived from it were cultivated on PDA or PDA supplemented with 100 ng/μL hygromycin B (Sigma, Sweden), respectively, at 28 °C. For all assays on solid media, inoculation was performed by transferring a 5 mm diameter agar plug of actively growing mycelium to the center of the Petri dish. Minimal liquid media (pH 4) supplemented with 0.5% (*w*/*v*) apple pectin (70–75% esterification; Sigma, Sweden), polygalacturonic acid sodium salt (Serva, Germany), sucrose or glucose prepared essentially as described by Shah et al. [23] were used for *C. rosea* and *T. reesei* biomass determination, enzyme activity assays and gene expression analysis unless otherwise specified. Biomass and fermentation broths were sampled after 72 h of cultivation at 200 rpm and 25 °C. *T. reesei* mutants were cultivated in liquid Mandels-Andreotti medium containing 5 g/L KH_2_PO_4_, 1.4 g/L (NH_4_)_2_SO_4_, 0.3 g/L Urea, 0.3 g/L MgSO_4_ × 7 H_2_O, 0.3 g/L CaCl_2_, 20 mL/L trace elements (250 mg/L FeSO_4_ x 7H_2_O, 85 mg/mL MnSO_4_ x H_2_O, 70 mg/mL ZnSO_4_ x 7H2O, 100 mg/mL CoCl_2_ x 2H_2_O), 2 g/L peptone from casein, 2 g/L Tween 20 supplemented with 1% of carbon source (glucose or apple pectin), pH adjusted to 5. For inoculation of liquid media, conidia were harvested from 7 days old Petri dishes in physiological solution with 0.025% of Tween 20, and 300 μL conidial suspension (2.5 × 10^8^ conidia/mL) were added into 125 mL of medium.

### Genome wide screening for PL1 homologues in *C. rosea* and Sordariomycetes

PL1 homologous proteins from 41 Sordariomycete species, *B. cinerea* and *S. sclerotiorum* (Leotiomycetes) (Additional file 1: Table S1) were identified by iterative BLAST approach and retrieved from the JGI DOE MycoCosm (http://genome.jgi.doe.gov) and NCBI (https://www.ncbi.nlm.nih.gov/genome/) genomic resource on 9th February 2015. The *C. rosea* protein sequences were trimmed for carbohydrate-binding module family 1 (CBM1) and carbohydrate esterases (CE) domains prior to BLAST analysis. The sequences with E-values higher than 1 × 10^− 8^ were not considered for the analysis. Conserved protein domains were predicted using NCBI Conserved Domain (CD) search [56], InterPro [57] and ExPASy ScanProsite [58] databases. The PL1 data set was unified and aligned using online multiple sequence alignment tool Clustal Omega [59]. Hierarchical clustering analysis of PL1 gene homologues in Sordariomycete and Leotiomycete species were performed using Statistica program (version 6.1; StatSoft Inc., Tulsa, OK) under complete linkage using Euclidean distances.

### Phylogenetic and protein sequence analyses

Predicted amino acid sequences were aligned by MUSCLE analysis tool [60] and were trimmed for the variable N- and C-terminal regions (signal peptide, CBM and CE domains). Phylogenetic analyses were performed using MrBayes v3.2.6 [61] using maximum parsimony optimality criterion and Bayesian inference of phylogeny, respectively. The Dayhoff amino acid substitution model was applied for Bayesian analysis and Metropolis-coupled Markov chain Monte Carlo (MCMCMC) sampling was performed with two simultaneous runs of four incrementally heated chains performed for 10 million generations. Trees were summarized after burning first 25% of the trees. Two completely independent analyses starting from different random trees were carried out. Bayesian posterior probabilities (PP) were obtained from the 50% majority-rule consensus of trees sampled every 100 generations after removing the first trees. PP values lower than 0.95 were not considered significant.

Regions of low amino acid conservation in PL1 protein alignments were identified by reverse conservation analysis (RCA), as described by Lee [62]. In short, Rate4Site (Version 2.01) was used to calculate the degree of conservation (S score, high scores correspond to low degree of conservation) for each amino acid position using the empirical Bayesian method [63, 64]. A sliding-window average (*n* = 7) of normalized S scores (mean was 0 and standard deviation was 1) was plotted in Excel (Microsoft) (W mean score) and significant peaks were defined by intensity (I) values of 0.5 [62].

### PEL12 homology modeling

A *C. rosea* PEL12 homology model was constructed by the I-TASSER server located at http://zhanglab.ccmb.med.umich.edu [65], with a C-score of − 0.45, an estimated TM score of 0.66 + − 0.13 and an estimated Root-Mean-Square Deviation (RMSD) of 7.4 + − 4.3 Å. I-TASSER utilizes a suite of predictive and modelling techniques like threading of the query sequence onto the closest known homologous protein crystal structures. The PEL12 model showed closest structural similarity, TM-score 0.89 and RMSD of 1.79, with the PL1 structure from *Acidovorax avenue* subsp. *citrulli* (PDB:ID 4HWV), a bacterial fruit and plant pathogen [29]. Figures and analysis were performed using the PyMOL Molecular Graphics System (Version 1.8 Schrödinger, LLC.) and pectin substrate and metal ligands were extracted from PDB:ID 3KRG [66].

### Deletion of the *pel12* gene in *C. rosea*

Multisite Gateway cloning system was used to generate the deletion construct as described previously [33, 34]. *C. rosea pel12* deletion mutants were generated by replacing *pel12* with the hygB selection cassette by homologous recombination using *Agrobacterium tumefaciens*-mediated transformation (ATMT) protocol for *C. rosea* [67]. Homologous integration of the deletion cassette in putative transformants was validated using a PCR screening approach as described in our previous work [33, 34] with primers located within the hygB cassette (Hyg F/Hyg R) together with primers located upstream (6915 KO F) and downstream (6915 KO R) of the construct (Additional file 1: Table S2). RT-PCR analysis was conducted on WT and deletion strains using RevertAid premium reverse transcriptase (Fermentas, St. Leon-Rot, Germany) and primer pairs specific for *pel12* to confirm the complete loss of *pel12* transcript in mutant strains.

### Heterologous expression of *C. rosea* PEL12 in *T. reesei*

Genomic DNA of *C. rosea* was isolated using a hexadecyltrimethylammonium bromide (CTAB)-based method [68]. Primers containing PacI and Xbal restriction sites were designed to target the 5′ and 3′ sides of the *pel12* gene (Additional file 1: Table S2). The *pel12* gene was amplified using Phusion DNA polymerase (Finnzymes, Vantaa, Finland) under the following conditions: initial denaturation for 60 s at 98 °C followed by 32 cycles of denaturation (10s at 98 °C), annealing (30s at 60 °C) and extension (72 °C for 20s). Final extension was performed for 5 min at 72 °C. The amplified fragment was then ligated with the pCR2.1-TOPO vector (Invitrogen) and the plasmid was transformed into One Shot TOP10 *Escherichia coli* competent cells using TOPO TA Cloning Kit (Invitrogen) according to manufacturer protocol. After the plasmid purification, *pel12* was restricted out of the TOP10 *pel12* plasmid using EcoRI and was digested by XbaI and PacI restriction enzymes (Fermentas). The pTreno_CBH1 vector [69], containing an enolase promoter, the *T. reesei cbh1* gene and *cbh2* terminator followed by the *hph* hygromycin resistance cassette, was also digested at the PacI and Xbal restriction sites and the *cbh1* gene was replaced by *pel12* using T4 DNA ligase kit (USB, Affymetrix)*.* The construct was amplified in *E. coli* and extracted using the Promega Midi Prep Kit (Sweden). The obtained plasmid was linearized with SbfI and transformed in electrocompetent *T. reesei* QM9414 spores following the electroporation protocol essentially as described by Linger et al. [69]. Hygromycin-resistant *pel12*OE transformants were checked for mitotic stability by several rounds of cultivations. Genomic DNA was extracted from *T. reesei* using the Qiagen DNeasy Plant Mini Kit following the manufacturer protocol. A PCR and quantitative PCR (qPCR) screening approach was performed to validate the integration of the pTreno_Tr*pel12* in transformants using 6915fwOE2/6915revOE2 primers as well as 6915_qPCR_fw2/6915_qPCR_rev2. *T. reesei* QM9414 and plasmid DNA were used as negative and positive controls, respectively.

### Protein detection and enzyme assays

*C. rosea* and *T. reesei* proteins secreted on 0.5% apple pectin, polygalacturonic acid, glucose or sucrose liquid medium ([23]; see above) were on-column concentrated using Vibraspin membrane columns with 10 kDa cut-off (Sartorius Stedim). For the protein separation on the SDS-PAGE gel, Laemmli sample buffer premixed with β-mercaptoethanol (9:1) was added to each sample in biological duplicates), mixed and denaturated for 3 min at 99 °C and applied to 4–20% pre-casted Mini Protein TGX stain-free SDS-PAGE gels (BioRad). The gels were stained using undiluted AcquaStain solution (Bulldog Bio). Distinct bands were excised, the proteins were reduced, alkylated and in-gel digested with trypsin according to a standard operating protocol. Thereafter, the samples were dried and resolved in 15 μl 0.1% formic acid. Peptides were separated in reversed-phase on a C18-column and electrosprayed on-line to Q Exactive Plus mass spectrometer (Thermo Finnigan). Tandem mass spectrometry (MS) was performed applying Higher-energy collisional dissociation (HCD) at the MS Facility, SciLifeLab, Uppsala University, Sweden. The database searches were performed using the Sequest algorithm embedded in Proteome Discoverer 1.4 (Thermo Fisher Scientific) towards predicted *C. rosea* protein models. The search criteria for protein identification were set to at least two matching peptides of 95% confidence level per protein.

Liquid state fermentations of *T. reesei* QM9414 and the *pel12*OE mutants were performed on minimal Mandels-Anderotti medium (see above). Fermentation was carried out in 500 mL Erlenmeyer flask in darkness at 28 °C, 220 rpm for 72 h. Samples were taken after 24, 48 and 72 h. Protein detection was performed using SDS-PAGE electrophoresis essentially as described above.

A standard pectate lyase kinetic assay was performed using culture filtrates from *T. reesei* WT and *pel12*OE mutants’ fermentations on 1% glucose essentially as described by Collmer et al. [12] with 0.24% (*w*/*v*) polygalacturonic acid mixed with 60 mM Tris-HCl and 0.6 mM CaCl_2_, pH 8.5 as substrate solution. 1 mL of crude supernatant was concentrated using Vivaspin columns with 10 kDa cutoff of polyethersulfan membrane (Sartorius, Germany) in a final volume of 100 μL. The fermentation broths of the mutants were further tested for pectin lyase activity using the same protocol by replacing polygalaturonic acid with apple pectin and omitting CaCl_2_. All enzyme activity assays were performed at pH 5 and 8.5. One unit of enzyme forms 1 μmol of 4,5-unsaturated product in 1 min under the conditions of the assay. The molar extinction coefficient for the unsaturated product at 232 nm is 4600 M^− 1^ cm^− 1^.

Assays with monosaccharides were conducted using 200 μL of *T. reesei* WT and *pel12*OE mutant culture filtrates grown for 72 h on 1% apple pectin as described above. The proteins were heat-inactivated at 90 °C for 15 min and separated by centrifugation. Arabinose, glucose, galactose, fructose, galacturonic acid and rhamnose in the supernatants were quantified by high-performance anion-exchange chromatography with pulsed amperometric gold electrode detection (HPAEC-PAD) paired with an anion exchange CarboPac PA1 column (4 × 250 mm plus 4 × 50 mm guard column) at 30 °C and 1 mL min^− 1^ flow rate. The initial mobile phase of 18 mM NaOH was held for 7 min; then a gradient from 18 mM to 119 mM NaOH mixed with 18 mM NaAc was applied for 11 min followed by a gradient to 100 mM NaOH mixed with 150 mM NaAc for 3 min. This concentration was held for 2.5 min followed by fast gradient to 200 mM NaOH which was maintained for further 3 min. Finally, 18 mM NaOH was applied for 15 min to equilibrate the columns for the next sample (20 μl injection volume). Ppm (parts per million) correspond to mg/l of the substrate detected.

### Phenotype microarrays

Growth rates on different carbon sources, except on apple pectin, were analyzed in biological duplicates using a phenotype microarray system for filamentous fungi (Biolog Inc., Hayward, CA), as described by Atanasova and Druzhinina [70]. Briefly, strains were cultivated on PDA for 7 days. Conidial inocula were prepared by rolling a sterile, wetted cotton swab over sporulating areas of the plates. The conidia were then suspended in sterile Biolog FF inoculating fluid (0.25% Phytagel, 0.03% Tween 40), gently mixed, and adjusted to a transmission of 75% at 590 nm (using a Biolog standard turbidimeter calibrated to the Biolog standard for filamentous fungi). A total of 90 μL of the conidial suspension was dispensed into each of the wells of the Biolog FF microplates (Biolog Inc.), which were incubated at 28 °C in darkness. The optical density (OD) at 750 nm (for detection of mycelial growth) was measured after 24, 48, 72, 96 and 120 h using a microplate reader (Biolog Inc.). Statistical analyses were performed using the Statistica software package (version 6.1; StatSoft Inc., Tulsa, OK). Growth rates on pectin were determined by cultivation of the fungi on solid Mandels-Anderotti minimal medium supplemented with 2% agarose and 1% apple pectin. The growth of mycelia was measured every 24 h.

### Detached leaf bioassay

A detached leaf assay for biocontrol of the grey mold pathogen B. cinerea was performed as described previously [33, 34]. In brief, leaves of 3-week-old *Arabidopsis thaliana* ecotype Colombia-0 (Col-0) plants, grown in a Percival growth chamber (CLF plant climates, GmbH, Germany) with growth conditions described before [71], were detached from plants and placed on water agar plate with the petiole inserted in the agar. A 5 μl droplet of conidial suspension (1 × 10^6^ conidia/mL) of *C. rosea* WT, *C. rosea* ∆*pel12* mutants, *T. reesei* WT and *T. reesei pel12*OE mutants were inoculated on the adaxial surface of the leaf, dried for 30 min and re-inoculated with equal conidial concentration of *B. cinerea* at the same place. Plates were kept in Percival growth chambers and high humidity was maintained by sealing the plates with parafilm. The diameter of necrotic lesions was measured post 56 h of inoculation under the microscope using a DeltaPix camera and software (DeltaPix, Denmark). Bioassay experiments were performed in 4 biological replicates and each replicate consisted of six leaves for each treatment. The experiment was repeated two times.

### Transcriptional profiling by reverse transcription qPCR

For gene expression analysis of *C. rosea* PL1 genes in different nutritional conditions (described above), mycelia were cultivated in liquid cultures as described by Shah et al. [23] and harvested 72 h post inoculation. For validation of *pel12* heterologous expression, *T. reesei* WT and *pel12*OE mutants were cultivated on minimal Mandels-Andreotti medium supplemented with 1% glucose or apple pectin (see above) for 48 h. Harvested biomass was immediately frozen in liquid nitrogen and stored at − 80 °C. RNA extraction from all samples was done using the Qiagen RNeasy kit following the manufacturer’s protocol (Qiagen, Hilden, Germany). RNA was treated with RNase-free DNaseI (Fermentas, St. Leon-Rot, Germany) and concentrations were determined using NanoDrop (Thermo Scientific, Wilmington, DE). One microgram of total RNA was reverse transcribed in a total volume of 20 μl using the iScript™ cDNA synthesis kit (BioRad, Germany). Transcript levels were quantified by qPCR using the EvaGreen qPCR Master Mix (Biotium) in an iQ5 qPCR System (Bio-Rad, Hercules, CA) using 98 °C for 2 min for initial denaturation and 40 cycles of 98 °C for 5 s, 58 °C for 10 s and 65 °C for 10 s. Melt curve analysis was performed after the qPCR reactions, to confirm that the signal was the result from a single product amplification. Relative expression levels for target genes in relation to actin expression [72, 73] were calculated from the Ct (cycle threshold) values and the primer amplification efficiencies by using the formula described by Pfaffl [74]. Gene expression analysis was carried out in three biological replicates, each based on three technical replicates. Primer sequences used for gene expression analysis are given in the Additional file 1: Table S2.

## Additional files

Additional file 1: **Table S1.** PL1 genes in to date available Sordariomycetes genomes together with plant pathogenic Leotiomycetes *Botrytis cinerea* and *Sclerotinia sclerotiorum*. For the presentation reasons the orthologues from Sordariomycetes, *B. cinerea* and *S. sclerotiorum* are assigned to one of the 17 *C. rosea* PL1 genes based on the highest E-value. Genome references: [24, 35, 36, 75–101]. **Table S2.** PCR and qPCR primers used in this study. PE annotate primer efficiency. attB and attBr site sequences are shown in lowercase. (XLSX 19 kb)

Additional file 2: **Figure S1.** Partial protein alignment of the closest neighbors of PEL12 and separation of the group A and B for further reverse conservation analysis. *C. rosea* paralogs BN869_T0006915 (PEL12) and BN869_T00007653 (PEL13) align to the group B, whereas the protein BN869_T0008859 (PEL2), *Fusaria* and some members of Sordariales are separated by several hallmarks forming the group A. **Figure S2.** SDS-PAGE gel showing the putative expression of the PEL12 protein (arrows) at 37 kDa in the fermentation broths of two independent *T. reesei* PEL12 overexpression strains *pel12*OE-1 and *pel12*OE-2. The strains were cultivated for 48 h in Mandels-Anderotti medium supplemented with 1% glucose. **Figure S3.** Carbon source utilization of *T. reesei* WT and *pel12*OE mutants on 95 different carbon sources based on BIOLOG Phenotypic assay for filamentous fungi. Mycelial density is shown for 96 h of incubation at 28 °C in darkness. Black, gray and white circles represent mycelial density of *T. reesei* QM9414 (wt) and the mutants *pel12*OE-1 and − 2, respectively. **Figure S4.** Validation of *pel12* mutant strains using PCR and RT-PCR. A: PCR verification of Δ*pel12* using primers located within the hygB cassette (Hyg F /Hyg R) in combination with primers located upstream and downstream regions from the deletion cassette (6915 ko F / 6915 ko R). A PCR product of ~ 2.5 kb using primers 6915 ko F / Hyg R and 6915 ko R / Hyg F were expected from a correct gene replacement. M, gene ruler DNA ladder mix; 1–3, independent Δ*pel12* mutants; 4, WT strain. B: RT-PCR analysis of *pel12* gene expression in WT and deletion strains using *pel12* specific primers. A PCR product of 92 bp was expected from the WT. M, gene ruler DNA ladder mix; 1, WT; 2–4, independent deletion strains. Primer combinations used for PCR and RT-PCR are given above the images. **Figure S5.** Carbon source utilization of *C. rosea* WT and ∆*pel12* mutants (∆*pel12* KOA, KOB, KOC) on 95 different carbon sources based on BIOLOG Phenotypic assay for filamentous fungi. Mycelial density is shown for 144 h of incubation at 28 °C in darkness. **Figure S6.** Measurement of *B. cinerea* necrotic lesions area on detached leaves of *Arabidopsis thaliana*. The leaves were inoculated with *C. rosea* strains (A) or *T. reesei* (B) strains spores 30 min before application of *B. cinerea* spores. Necrotic lesion area was measured under the microscope using a DeltaPix camera and software 56 h post inoculations. Only *B. cinerea* inoculated leaves were used as control. Error bars represent standard deviation based on three biological replicates. Different letters indicate statistically significant differences (*P* ≤ 0.05) based on the Fisher test. (PDF 633 kb)

## Abbreviations

(NH_4_)_2_SO_4_: ammonium sulfate
ATMT: *Agrobacterium tumefaciens*-mediated transformation
BLAST: Basic Local Alignment Search Tool
Ca^2+^: calcium ion
CaCl_2_: calcium chloride
CAZy: Carbohydrate-Active enZYmes
CBM: Carbohydrate-Binding Module
CD: Conserved Domain
CE: Carbohydrate Esterases
CoCl_2_: cobalt(II) chloride
Ct: Cycle threshold
CTAB: hexadecyltrimethylammonium bromide
DNA: deoxyribonucleic acid
FeSO_4_: iron(II) sulfate
H_2_O: water
HCD: Higher-energy Collisional Dissociation
HPAEC-PAD: High-Performance Anion-Exchange Chromatography with Pulsed Amperometric gold electrode
JGI DOE: Joint Genome Institute of the United States Department of Energy
KH_2_PO_4_: monopotassium phosphate
MCMCMC: Metropolis-coupled Markov chain Monte Carlo
MgSO_4_: magnesium sulfate
MnSO_4_: manganese(II) sulfate
MS: Mass Spectrometry
NaAc: sodium acetate
NaOH: sodium hydroxide
NCBI: National Center for Biotechnology Information
OD: Optical Density
OE: overexpression
PCR: Polymerase Chain Reaction
*pel*: pectate lyase gene
PEL: pectate lyase protein
*pel12*OE: *pel12* overexpression mutants
PL: Polysaccharide Lyase
Ppm: Parts per million
RCA: Reverse Conservation Analysis
RMSD: Root-Mean-Square Deviation of atomic positions
RNA: Ribonucleic acid
RT-PCR: Reverse Transcription PCR
SDS-PAGE: Sodium Dodecyl Sulfate Polyacrylamide Gel Electrophoresis
Tris-HCl: Trisaminomethane hydrochloride
WT: Wild Type
ZnSO_4_: zinc sulfate
Δ*pel12*: *pel12* deletion mutants
