# Supplementary material for: Evolution and functional characterization of pectate lyase PEL12, a member of a highly expanded Clonostachys rosea polysaccharide lyase 1 family
Source: BMC Microbiol. 2018 Nov 7;18:178. doi: 10.1186/s12866-018-1310-9 (PMC6223089; doi:10.1186/s12866-018-1310-9)

Additional figure 1

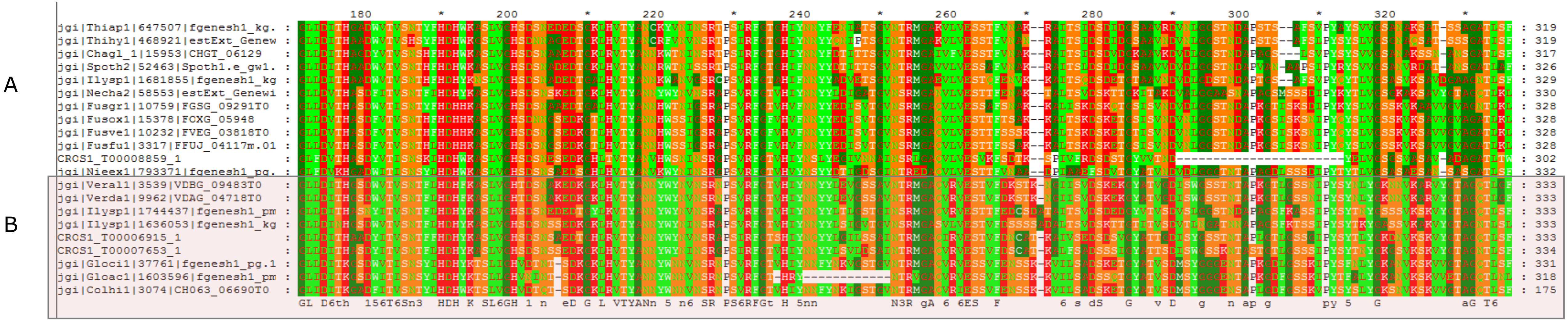

Additional figure 2

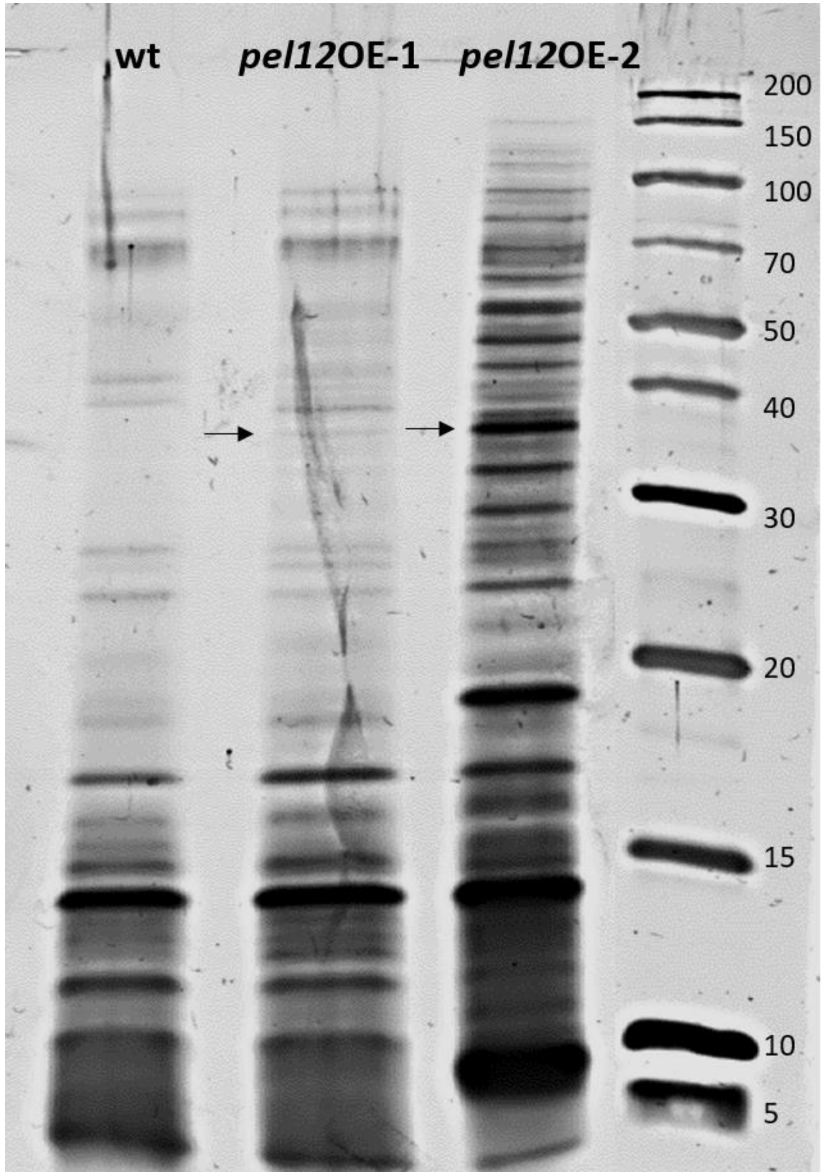

Additional figure 3

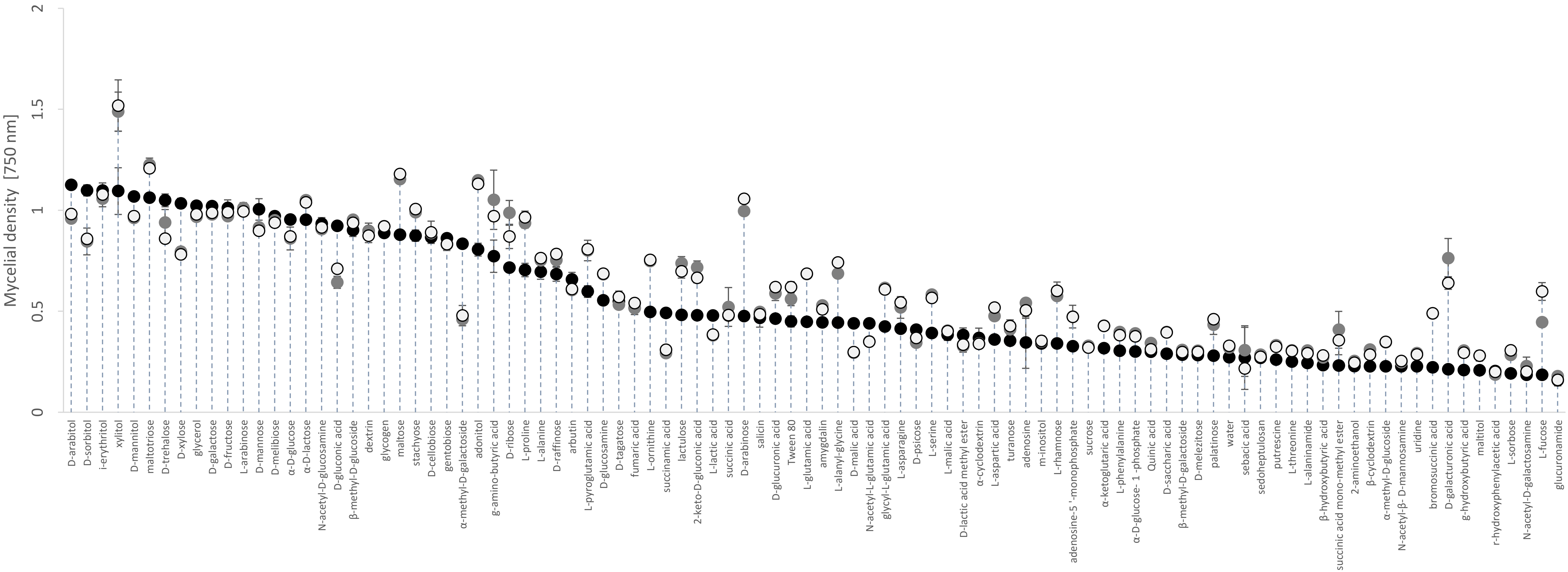

Additional figure 4

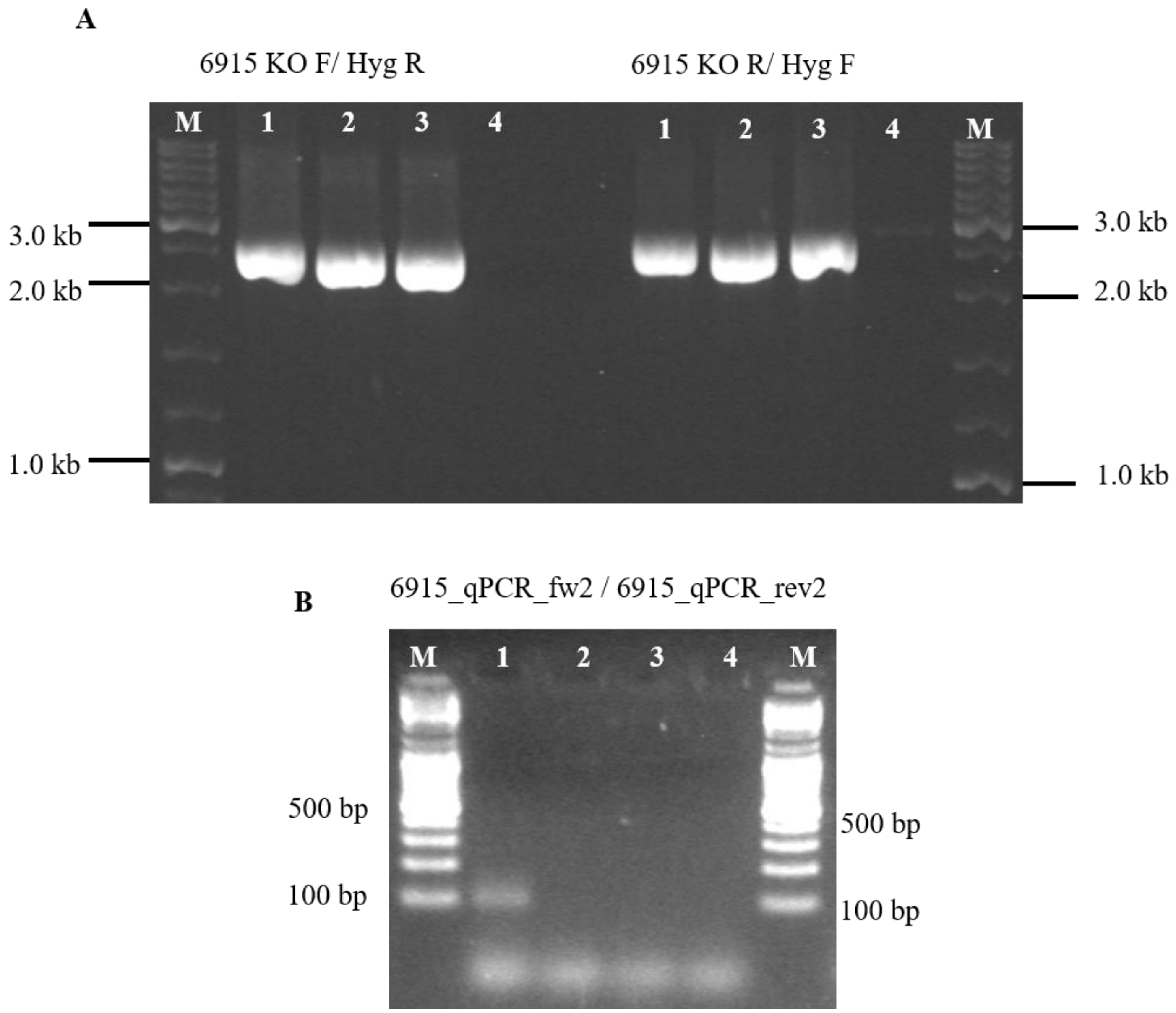

Additional figure 5

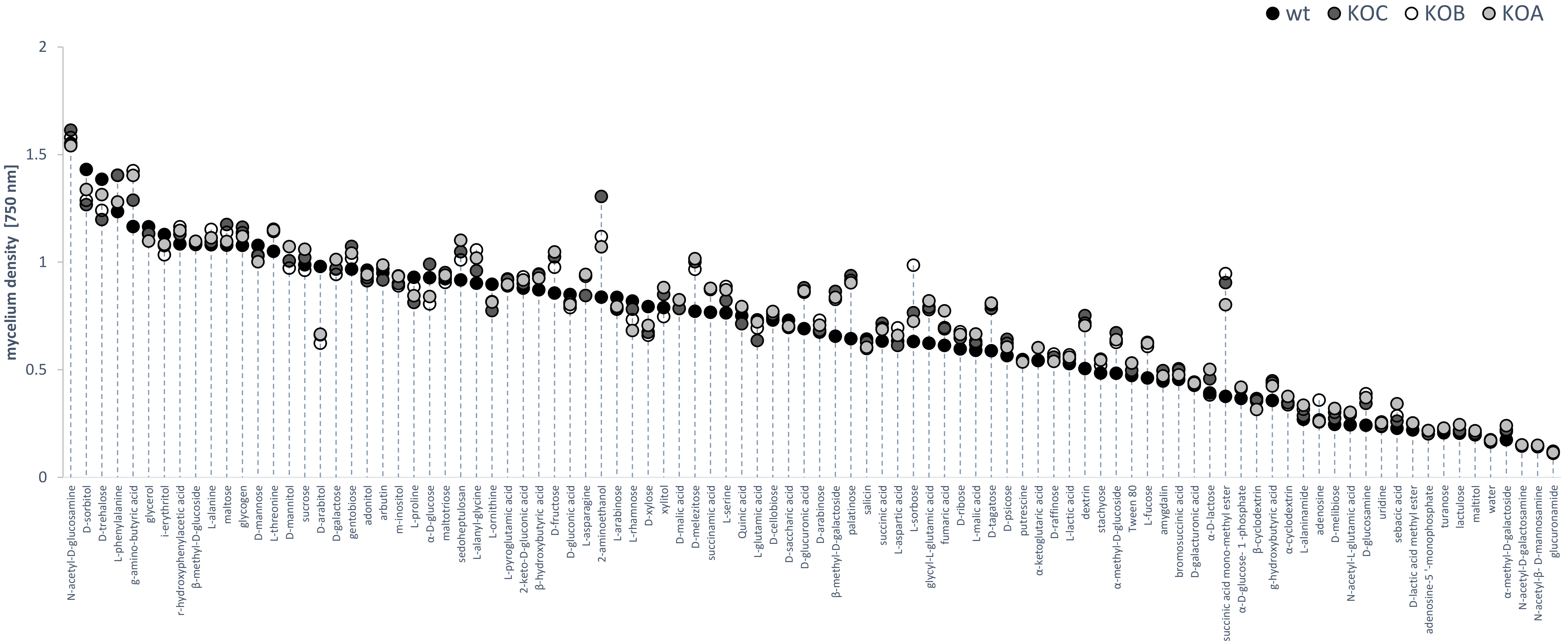

Additional figure 6

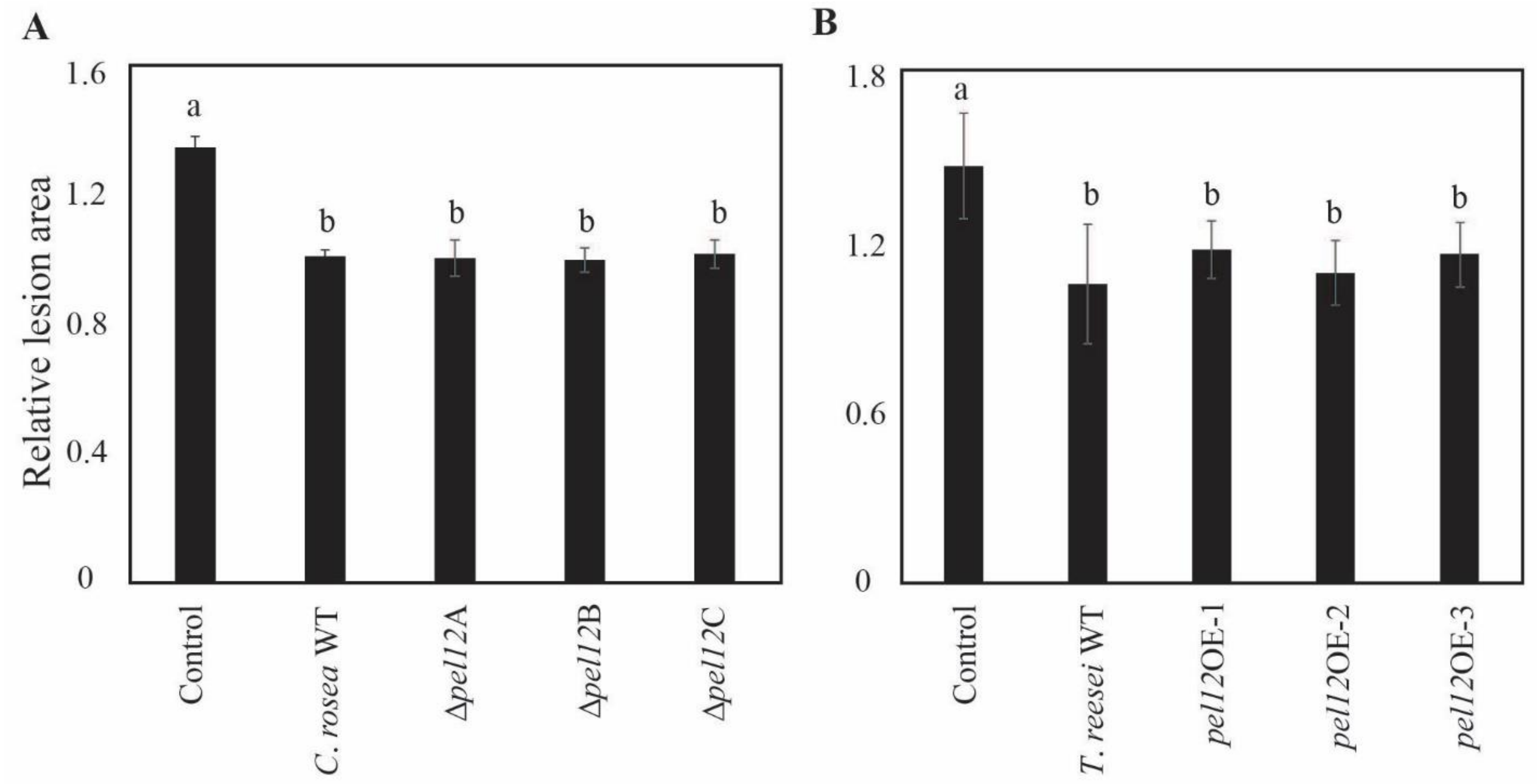

Supplement: Supplementary file 2 — Figure S1. Partial protein alignment of the closest neighbors of PEL12 and separation of the group A and B for further reverse conservation analysis. C. rosea paralogs BN869_T0006915 (PEL12) and BN869_T00007653 (PEL13) align to the group B, whereas the protein BN869_T0008859 (PEL2), Fusaria and some members of Sordariales are separated by several hallmarks forming the group A. Figure S2. SDS-PAGE gel showing the putative expression of the PEL12 protein (arrows) at 37 kDa in the fermentation broths of two independent T. reesei PEL12 overexpression strains pel12OE-1 and pel12OE-2. The strains were cultivated for 48 h in Mandels-Anderotti medium supplemented with 1% glucose. Figure S3. Carbon source utilization of T. reesei WT and pel12OE mutants on 95 different carbon sources based on BIOLOG Phenotypic assay for filamentous fungi. Mycelial density is shown for 96 h of incubation at 28 °C in darkness. Black, gray and white circles represent mycelial density of T. reesei QM9414 (wt) and the mutants pel12OE-1 and − 2, respectively. Figure S4. Validation of pel12 mutant strains using PCR and RT-PCR. A: PCR verification of Δpel12 using primers located within the hygB cassette (Hyg F /Hyg R) in combination with primers located upstream and downstream regions from the deletion cassette (6915 ko F / 6915 ko R). A PCR product of ~ 2.5 kb using primers 6915 ko F / Hyg R and 6915 ko R / Hyg F were expected from a correct gene replacement. M, gene ruler DNA ladder mix; 1–3, independent Δpel12 mutants; 4, WT strain. B: RT-PCR analysis of pel12 gene expression in WT and deletion strains using pel12 specific primers. A PCR product of 92 bp was expected from the WT. M, gene ruler DNA ladder mix; 1, WT; 2–4, independent deletion strains. Primer combinations used for PCR and RT-PCR are given above the images. Figure S5. Carbon source utilization of C. rosea WT and ∆pel12 mutants (∆pel12 KOA, KOB, KOC) on 95 different carbon sources based on BIOLOG Phenotypic assay for filamentous fungi [file 12866_2018_1310_MOESM2_ESM.pdf]
